# A Novel Proposal for Classifying Pediatric Tonsillectomy Complications: Modified Clavien Classification

**DOI:** 10.7759/cureus.8701

**Published:** 2020-06-19

**Authors:** Omer Hizli, Serkan Kayabasi, Bugra Cengiz, Aydin Acar

**Affiliations:** 1 Otolaryngology, Prof. Dr. A. Ilhan Ozdemir Education and Research Hospital, Giresun University, Giresun, TUR; 2 Otolaryngology, Faculty of Medicine, Aksaray University, Aksaray, TUR; 3 Otorhinolaryngology, Bagcilar Trainining and Research Hospital, İstanbul, TUR; 4 Otolaryngology, Kecioren Education and Research Hospital, Ankara, TUR

**Keywords:** tonsillectomy, complications, classification, pediatric

## Abstract

Introduction

Tonsillectomy is one of the most common operations performed in the otolaryngology practice and there does not exist a systematic classification for tonsillectomy complications in the prior literature. In this study, we aimed at presenting a novel classification system to the current literature and analyzing complications of pediatric tonsillectomy based on this novel classification system.

Methods

A novel classification system based on modified Clavien classification was constituted for pediatric tonsillectomy complications. Medical records of 534 patients underwent tonsillectomy were retrospectively investigated and complication rates of tonsillectomy between children and adults were compared using this classification

Results

In total, 454 pediatric patients (258 males and 196 females, age range = 3-17 years) who underwent cold-knife tonsillectomy were eligible for the study. To compare the complication rates of the pediatric patients with adults, 80 adults with tonsillectomy (50 males and 30 females, age range 18-46) were also included. In children, the most common complication was dehydration, seen in 13 (2.86%) patients. The most serious complication was tooth aspiration (Grade 4a), seen in only one (0.22%) patient. Fifteen (3.3%) pediatric patients experienced more than one complication. Overall complication rate of pediatric tonsillectomy was 10.13% (46 patients). In adults, the most common complication was postoperative bleeding, seen in 11 (13.75%) adult patients. The most serious complication was Grade 3a postoperative bleeding, seen in four (5%) patients. Overall complication rate of adult tonsillectomy was 21.25% (17 patients). Overall complication rate of pediatric tonsillectomy was significantly lower compared with the complication rate of adult tonsillectomy (10.13% vs. 21.25%, p = 0.004, X^2 ^= 8.07).

Conclusion

Modified Clavien classification is a novel and simple tool to analyze and categorize complications of pediatric tonsillectomy.

## Introduction

Tonsillectomy and adenotonsillectomy are two of the most often performed operations in otolaryngology practice [1]. Current indications of tonsillectomy consist of recurrent tonsillitis, tonsillar hypertrophy resulting in apnea and the suspicion of malignancy [2]. Tonsillectomy seems as a safe procedure in the pediatric population, while there exists a potential for significant morbidity and even mortality in respect of complications in the adult population. Complications of tonsillectomy may occur within the first postoperative 24 hours, and weeks or even months later [3]. Postoperative complications such as fever, painful swallowing, ear pain, dehydration, and uvula edema may prolong the healing process. More serious, life-threatening conditions are the complications associated with the airway and general anesthesia, including aspiration risk, pulmonary edema, embolism, atlantoaxial subluxation, eustachian injury, nasopharyngeal stenosis, and velopharyngeal failure [4]. Rates of hemorrhage following tonsillectomy ranged from 0.3% to 13.9% in the prior literature, and the average was determined as 4.5% [5]. Complications following tonsillectomy are known to have a psychological effect on the patient, their family, and the physician and entail a financial burden. Therefore, the potential complications that can develop following tonsillectomy are of the greatest importance to otolaryngology physicians [6]. The aim of this study was to present a novel classification system for the complications of tonsillectomy in pediatric patients and to compare the complication rates of the pediatric tonsillectomy with adults.

## Materials and methods

Patients, the modified Clavien classification system and study design

Approval for this retrospective study was provided by the Local Ethics Committee and all procedures were performed in accordance with the principles of the Helsinki Declaration. Before compiling the retrospective data, all tonsillectomy complications previously reported in the literature and all potential complications were reviewed, and categorized based on the modified Clavien classification system and a novel classification for tonsillectomy complications was constituted by the Author Hizli (Table 1) [3,4,7-10].

**Table 1:** Modified Clavien classification system of tonsillectomy complications Suffix ‘d’: for complications requiring long-term follow-up, e.g., 3bd, 1d, 2d, 4ad…

GRADES OF THE COMPLICATIONS
GRADE 1. Deviation from the normal course without the need for additional pharmacological treatment or surgical-endoscopic-radiological intervention. Antiemetics, antipyretics, analgesics, diuretics, electrolyte supplementation and physiotherapy/speech therapy are allowed to recover the complication. Postoperative bleeding that controlled by bedside treatment; postoperative pain requiring additional analgesics or causing swallowing disorder; dental, alveolar, oral mucosal, uvular, lingual, palatal mild injuries; cautery burns (the wide burns requiring long term follow-up takes the suffix ‘d’) accidental extubation; hypoglycemia, hyponatremia, dehydration, nausea- vomiting; uvula or tongue edema; taste disorder; postoperative fever; neck pain, torticollis; atelectasis, subcutaneous emphysema; atlantoaxial subluxation; postoperative otitis media with effusion; laryngospasm without any treatment requirement; cervical vertebral osteoarthritis; rhinolalia and hypernasality; arrhythmia and vagal stimulation without need for additional medication; lingual or glossopharyngeal nerve paralysis (takes the suffix 'd'); vagal injury without need for medication for parasympathetic activation (takes the suffix 'd'); Hypoglossal nerve injury without speech or swallowing disorder (takes the suffix 'd'); Uvula amputation or necrosis (takes the suffix 'd'); nasopharyngeal/hypopharyngeal stenosis without any surgery requirement (takes the suffix 'd'); velopharyngeal insufficiency without any surgery requirement (takes the suffix 'd').
GRADE 2. Complications requiring pharmacological treatment other than allowed for Grade-1 complications like blood transfusion. Postoperative bleeding controlled by bedside treatment, but required blood transfusion; temporomandibular joint dislocation (intraoperatively replaced); mediastinal emphysema; laryngospasm requiring additional medication; arrhythmia and vagal stimulation requiring additional medication; infections requiring antibiotics (bronchitis, pneumonia, otitis media, peritonsillar or retropharyngeal abscess, deep neck infections etc.); Epileptic convulsions, schizoid reactions; vagal injury requiring medication for parasympathetic activation (takes the suffix 'd'); severe hypoglossal nerve injury causing speech or swallowing disorder (takes the suffix 'd'); anorexia (takes the suffix 'd').
GRADE 3. Complications requiring surgery, endoscopic or radiological intervention. 3a: Without general anesthesia: Postoperative bleeding controlled in the operating room; temporomandibular joint dislocation (postoperatively replaced); peritonsillar abscess; pneumothorax. 3b: Under general anesthesia: Postoperative bleeding controlled in operating room requiring exploration; temporomandibular joint dislocation (postoperatively replaced); deep neck infections; pulmonary abscess nasopharyngeal/ hypopharyngeal stenosis (take the suffix 'd'); velopharyngeal insufficiency (take the suffix 'd').
GRADE 4. Life-threatening, serious complications requiring intensive care. 4a: Single system dysfunction: Blood or foreign body aspiration; meningitis or brain abscess; pulmonary edema, insufficiency or embolism; subacute bacterial endocarditis; cardiac insufficiency; renal insufficiency cerebral infarct; blindness or deafness (take the suffix 'd'); 4b: Multi-system dysfunction: Coexistence of 4a complications; sepsis; septic shock. 4c: Life-threatening emergencies: Carotid artery injury, hypovolemic shock; cardiac arrest; cavernous sinus thrombophlebitis; bulbar poliomyelitis; anaphylaxis; malignant hyperthermia; life-threatening vagal stimulation and shock
GRADE 5. Death of the patient (not related to primary disease like tonsillar carcinoma)

The medical records of 534 patients (454 pediatric patients, and 80 adult patients as control group) undergone tonsillectomy for various indications between January 2012 and January 2020 were examined. The age, gender, intraoperative and postoperative complications, the severity of complications and additional diseases were retrospectively retrieved from the medical records of the patients (according to Table 1). The patients who underwent surgery had normal values of prothrombin time and active partial prothrombin time measured in the complete blood count preoperatively.

The study included only the patients who underwent conventional cold-knife tonsillectomy. During the operation, bleeding control was carried out using bipolar cautery in all patients. Postoperative follow-up was monitored for one day in the hospital, then patients were discharged on the following day. Adult patients received 2 x 1000 mg/day amoxicillin-clavulanate tablets for 10 days postoperatively and pediatric patients received 2 x 25 mg/kg/day amoxicillin-clavulanate suspension for 10 days. The patients who underwent tonsillectomy with a method other than the cold-knife technique, with another surgical procedure in the same session, as a part of multi-level surgery for obstructive sleep apnea and the patients with suspicion of malignancy were excluded from the study.

The classification system on which our study was based was determined in 1992 by Clavien et al. to evaluate the complications of surgery formed by general surgery [10]. This classification was modified by Dindo et al. in 2004 because of the lack of consideration of hospital stay, life-threatening complications, and the lack of assessment of permanent complications [11]. Tonsillectomy complications were classified into five grades based on the modified Clavien classification system. Grade 1 was described as a deviation from the normal course without the need for additional pharmacological treatment or surgical-endoscopic-radiological intervention. Grade 2 was the complications requiring pharmacological treatment other than allowed for Grade-1 complications like blood transfusion. Grade 3 complications were the ones requiring surgery, endoscopic or radiological intervention (3a without general anesthesia, 3b under general anesthesia). Life-threatening, serious complications requiring intensive care including single system dysfunction (4a), multisystem dysfunction (4b), and emergencies (4c) were classified as Grade 4. The death of the patient was considered as Grade 5 complication. Finally, any complication requiring long-term follow-up took the suffix “d” (Table 1).

Statistical analysis

The results are presented as number (percentage) using descriptive tables. To compare the complication rates of the pediatric patients with adults, we used Chi-square test. For statistical analysis, we used Statistical Package for the Social Sciences (SPSS) 23.0 software for Windows (SPSS Inc., Chicago, IL). A p-value under 0.05 was considered statistically significant.

## Results

In total, 454 pediatric patients (258 males and 196 females, age range = 3-17 years) underwent conventional cold-knife tonsillectomy were eligible for the study. To compare the complication rates of the pediatric patients with adults, 80 adults (50 males and 30 females, age range = 18-46 years) underwent conventional cold-knife tonsillectomy were also included as a control group (Table 2).

**Table 2:** Demographic features of pediatric and adult patients

	Pediatric Patients	Adult Patients	Total
Male	258 (48.3%)	50 (9.37%)	308 (57.67%)
Female	196 (36.71%)	30 (5.62%)	226 (42.33%)
Total	454 (85.01%)	80 (14.99%)	534 (100%)

The distribution of tonsillectomy complications in pediatric patients according to the modified Clavien classification system was presented in Table 3.

**Table 3:** Distribution of pediatric tonsillectomy complications (n = 454) *The total number of the pediatric patients experienced at least one complication, since 15 patients experienced more than one complication

Grade	Complication	Frequency
Grade 1	Postoperative bleeding	4 (0.88%)
	Postoperative pain- swallowing disorder	4 (0.88%)
	Dehydration (with nausea- vomiting)	13 (2.86%)
	Hypoglycemia	2 (0.44%)
	Dental injury	2 (0.44%)
	Postoperative fever	6 (1.32%)
	Arrhythmia	1 (0.22%)
	Neck pain	1 (0.22%)
	Uvula edema	3 (0.66%)
	Otitis media with effusion	1 (0.22%)
	Oral mucosal injury	1 (0.22%)
	Torticollis	1 (0.22%)
	Rhinolalia and hypernasality	1 (0.22%)
Grade 1d	Uvula amputation	1 (0.22%)
	Velopharyngeal insufficiency	4 (0.88%)
	Nasopharyngeal stenosis	1 (0.22%)
Grade 2	Postoperative bleeding	1 (0.22%)
	Laryngospasm	3 (0.66%)
	Postoperative infection	2 (0.44%)
	Temporomandibular dislocation	1 (0.22%)
Grade 2d	Anorexia	1 (0.22%)
Grade 3a	Postoperative bleeding	3 (0.66%)
	Peritonsillar abscess	1 (0.22%)
Grade 3b	Postoperative bleeding	2 (0.44%)
Grade 4a	Tooth aspiration	1 (0.22%)
Total		46* (10.13%)

According to this analysis, the most common complication was dehydration, seen in 13 (2.86%) pediatric patients. The most serious complication was tooth aspiration (Grade 4a), seen in only one (0.22%) pediatric patient. Fifteen (3.3%) pediatric patients experienced more than one complication. Overall complication rate of pediatric tonsillectomy was 10.13% (46 patients).

The distribution of tonsillectomy complications in adults according to the modified Clavien classification system was presented in Table 4.

**Table 4:** Distribution of adult tonsillectomy complications (n = 80)

Grade	Complication	Frequency
Grade 1	Postoperative bleeding	4 (5%)
	Postoperative pain-swallowing disorder	1 (1.25%)
	Neck pain	1 (1.25%)
	Taste disorder	2 (2.5%)
Grade 2d	Anorexia	2 (2.5%)
Grade 3a	Postoperative bleeding	4 (5%)
Grade 3b	Postoperative bleeding	3 (3.75%)
Total		17 (21.25%)

According to this analysis, the most common complication was postoperative bleeding, seen in 11 (13.75%) adult patients. The most serious complication was Grade 3a postoperative bleeding, seen in four (5%) adult patients. Overall complication rate of adult tonsillectomy was 21.25% (17 patients). Overall complication rate of pediatric tonsillectomy was significantly lower than the complication rate of adult tonsillectomy (10.13% vs. 21.25%, p = 0.004, X^2 ^= 8.07). However, pediatric patients experienced more varied types of complications in a wider grade range compared with adults.

## Discussion

In this study, we presented a novel classification system for pediatric tonsillectomy complications that was constituted based on the prior complication grading system modified by Dindo et al. [11]. In 1992, Clavien et al. reported a grading system for surgical complications, mainly focusing on life-threatening complications and long-term disability [10]. They also confirmed their classification in a survey of 6336 patients and this new grading system was a reliable tool for quality assessment in surgery to be used worldwide [11]. In the prior literature, many tonsillectomy complications from the large series of various centers were reported; however, there does not exist a clear consensus on how to deal and categorize these complications because of the lack of a standardized classification system [3,4,6]. Importantly, in this context, a standardization is required both to categorize complications systematically and inform the patients, instead of classifying complications as the only minor to major. Like the classification system modified previously, we analyzed every single potential complication of tonsillectomy, and categorized them based on the severity, the need for additional medication, the need for intervention or surgery and characteristics of the complication (such as being a cause of organ dysfunction or a life-threatening emergency) [10,11]. Thus, any complication such as postoperative bleeding or temporomandibular joint dislocation could be categorized in more than one grades, since postoperative bleedings requiring blood transfusion were categorized as Grade 2 while bleedings requiring surgical intervention under general anesthesia were categorized as Grade 3b. Classifying the complications using our novel system would provide a rapid evaluation opportunity for further investigations and this system would be an overcoming of an important shortcoming in this field in the literature.

From the medical archive of our institution, we compiled the data of 534 patients who underwent conventional cold-knife tonsillectomy and recorded the grade of every single complication. The complication rate of pediatric patients (10.13%) was significantly lower compared to the adults (21.25%). However, pediatric patients experienced more varied types of complications in a wider grade range compared with adults. The most serious complication in pediatric patients was tooth aspiration causing mild breathing disorder, and the grade of this complication was 4a. Aspiration of foreign bodies including dislodged tooth, forgotten swabs and blood may be encountered during/after surgery, and it may result in airway obstruction, even may require bronchoscopy or tracheotomy in the operating room, accordingly, we categorized the foreign body aspiration as Grade 4a in our classification system [8,12]. In adults, the highest grade of all complications was 3b, and it was postoperative bleeding requiring an intervention under general anesthesia. Additionally, postoperative bleeding was the most common complication in adults. Consequently, we can state that postoperative bleeding is a more remarkable and serious complication in adults rather than children. Dehydration accompanied by nausea and/or vomiting was the most common complication (2.86%) in our pediatric population. Postoperative pain causing swallowing disorder and malnutrition, nausea and vomiting after tonsillectomy is not rare, and may result in dehydration in the pediatric population. Dehydration was also reported as a reason for a hospital revisit after tonsillectomy [13]. In our classification system, we categorized dehydration as Grade 1, because electrolyte supplementation is an allowed therapy for Grade 1 complications. Thus, early treatment of dehydration is crucial since untreated dehydration may result in hypovolemic shock that categorized as Grade 4c complication as being a life-threatening emergency.

In our pediatric population, four (0.88%) patients experienced velopharyngeal insufficiency without any need of surgery (Grade 1d) and one of these patients had Trisomy 21 (Down syndrome). In addition, all patients underwent adenoidectomy along with tonsillectomy. Velopharyngeal insufficiency may also be encountered after tonsillectomy alone, but it is more common in the pediatric population because tonsillectomy is generally performed along with adenoidectomy in children [14]. Consistently, no patient experienced a velopharyngeal insufficiency in our adult population. Moreover, a pediatric patient with Trisomy 21 might be complicated with atlantoaxial subluxation (a Grade 1 complication treated with neck stabilization and analgesics), but we did not encounter atlantoaxial subluxation in our study population.

One of the remarkable complications was a temporomandibular joint dislocation, seen only in one pediatric patient (0.88%). This complication may be encountered during many surgical procedures depending on the endotracheal intubation process, even during endoscopy, but temporomandibular joints of the patients undergoing tonsillectomy might be more likely to be dislocated because of the impact of the mouth retractor [15-17]. The dislocation of our patient was diagnosed in the operating room, and a replacement was performed before awakening. Thus, the complication was categorized as Grade 2. If it had not been diagnosed before awakening, the patient might have required an additional intervention under general anesthesia, meaning that the complication would have been categorized as 3b. Hence, surgeons should examine the temporomandibular joints of the patients before awakening after tonsillectomy, to avoid a 3b complication.

Although presenting a novel classification system to the current literature, this study has several limitations. We included 454 pediatric patients but including only 80 adults in the study might be considered as insufficient to reach accurate results. However, tonsillectomy (along with adenoidectomy) is mainly a pediatric surgery and we naturally had fewer adult patients. Additionally, we utilized our novel classification system only on a retrospective data, hence further prospective investigations tracking, recording, and grading every single complication during/after tonsillectomy might provide more accurate results on the complication prevalence.

## Conclusions

Modified Clavien classification system is a novel and simple tool to analyze and categorize the complications of pediatric tonsillectomy. The complication rate of pediatric tonsillectomy was significantly lower compared with the complication rate of adult tonsillectomy (10.13% vs. 21.25%). However, pediatric patients experienced complications in more varied grades compared to adults.
